# Supplementary material for: Role and mechanism of NCAPD3 in promoting malignant behaviors in gastric cancer
Source: Front Pharmacol. 2024 Apr 22;15:1341039. doi: 10.3389/fphar.2024.1341039 (PMC11070777; doi:10.3389/fphar.2024.1341039)
Supplement: Supplementary file 11 [file DataSheet2.ZIP › GSEA/GO_CC/my_analysis.Gsea.1599461408869/GO_PRERIBOSOME.html]

Details for gene set GO\_PRERIBOSOME[GSEA]

|  || Dataset | filtered\_dataset.sample\_info.cls#WT\_versus\_NCAPD3\_MUT |
| Phenotype | sample\_info.cls#WT\_versus\_NCAPD3\_MUT |
| Upregulated in class | WT |
| GeneSet | GO\_PRERIBOSOME |
| Enrichment Score (ES) | 0.3808407 |
| Normalized Enrichment Score (NES) | 1.6953307 |
| Nominal p-value | 0.032367975 |
| FDR q-value | 0.3401395 |
| FWER p-Value | 0.723 |
Table: GSEA Results Summary

  

Fig 1: Enrichment plot: GO\_PRERIBOSOME      
 Profile of the Running ES Score & Positions of GeneSet Members on the Rank Ordered List

  

| SYMBOL | TITLE | RANK IN GENE LIST | RANK METRIC SCORE | RUNNING ES | CORE ENRICHMENT || 1 | 8568 | RRP1 | 22 | 1.027 | 0.0733 | Yes |
| 2 | 55781 | RIOK2 | 135 | 0.763 | 0.0587 | Yes |
| 3 | 51018 | RRP15 | 192 | 0.685 | 0.0778 | Yes |
| 4 | 9724 | UTP14C | 301 | 0.591 | 0.0511 | Yes |
| 5 | 84946 | LTV1 | 319 | 0.583 | 0.0895 | Yes |
| 6 | 26156 | RSL1D1 | 332 | 0.574 | 0.1306 | Yes |
| 7 | 10885 | WDR3 | 430 | 0.515 | 0.1053 | Yes |
| 8 | 27340 | UTP20 | 455 | 0.501 | 0.1315 | Yes |
| 9 | 10969 | EBNA1BP2 | 483 | 0.485 | 0.1541 | Yes |
| 10 | 84261 | FBXW9 | 491 | 0.477 | 0.1905 | Yes |
| 11 | 51388 | NIP7 | 593 | 0.428 | 0.1548 | Yes |
| 12 | 54663 | WDR74 | 628 | 0.408 | 0.1656 | Yes |
| 13 | 23212 | RRS1 | 681 | 0.387 | 0.1617 | Yes |
| 14 | 10607 | TBL3 | 698 | 0.380 | 0.1831 | Yes |
| 15 | 23076 | RRP1B | 709 | 0.370 | 0.2080 | Yes |
| 16 | 55759 | WDR12 | 731 | 0.359 | 0.2241 | Yes |
| 17 | 134430 | WDR36 | 733 | 0.359 | 0.2545 | Yes |
| 18 | 51602 | NOP58 | 750 | 0.348 | 0.2732 | Yes |
| 19 | 51118 | UTP11L | 773 | 0.336 | 0.2865 | Yes |
| 20 | 51154 | MRTO4 | 794 | 0.327 | 0.3004 | Yes |
| 21 | 23246 | BOP1 | 797 | 0.325 | 0.3272 | Yes |
| 22 | 27341 | RRP7A | 801 | 0.320 | 0.3528 | Yes |
| 23 | 91695 | RRP7BP | 802 | 0.320 | 0.3806 | Yes |
| 24 | 10528 | NOP56 | 837 | 0.285 | 0.3808 | Yes |
| 25 | 8780 | RIOK3 | 948 | -0.367 | 0.3333 | No |
Table: GSEA details [plain text format]

  

Fig 2: GO\_PRERIBOSOME      
 Blue-Pink O' Gram in the Space of the Analyzed GeneSet

  

Fig 3: GO\_PRERIBOSOME: Random ES distribution      
 Gene set null distribution of ES for **GO\_PRERIBOSOME**
